# Factors affecting the active aging situation in Bangladesh

**DOI:** 10.3389/fpubh.2025.1517482

**Published:** 2025-03-06

**Authors:** Sadiya Afrin, Md. Mehedi Hasan Khan, Md. Aminul Haque

**Affiliations:** Department of Population Sciences, University of Dhaka, Dhaka, Bangladesh

**Keywords:** assessment, determinants of active aging, active aging, Bangladesh, factors of aging

## Abstract

**Background:**

The older population in Bangladesh is growing rapidly, from 8.0% in 2020 to 22.0% in 2050. However, the determinants of active aging are scarcely known.

**Objective:**

The study aimed to assess the determinants influencing the active aging situation in Bangladesh.

**Methods:**

A cross-sectional study was conducted among 518 older adults aged 60 and over. Following the WHO active aging model, the respondents’ socio-demographic, personal, behavioral, and physical environment and health and social services characteristics were collected using a semi-structured questionnaire. Multiple linear regression was performed to assess the effect of the determinants on active aging, followed by the bivariate level of analysis.

**Results:**

The determinants of active aging were deeply rooted in the respondents’ socio-cultural, economic, and spatial conditions. Nine out of 23 determinants, like marital status, income, decision-making capacity, regular walking/physical exercise, smokeless tobacco consumption, newspaper reading as a leisure activity, use of medicine, and health service accessibility, significantly influence active aging. The active aging score was 10–15% higher among the respondents who regularly adhered to the above determinants.

**Conclusion:**

Effective initiatives are needed to improve the socio-cultural, economic, and health system-related determinants of active aging to enhance the active aging situation. Concerned bodies of the country, ministries, departments, and development partners should take appropriate measures to increase awareness and the participation of people in lifestyle-related determinants to improve the active aging situation in the country.

## Introduction

Population aging is a major global trend that impacts all countries at different rates and levels (1). The number of people aged 60 years and over worldwide was projected to grow from 901 million in 2015 to 2.1 billion by 2050 (2). Population aging is a global phenomenon that requires action at the international, national, regional, and local levels (3).

In Bangladesh, the population aged 60 years and over has risen from 7.7% in 2015 to 9.5% in 2023 (4), with the country’s increasing life expectancy from 57.5 in 1990 to 72.3 in 2023 (4, 5). It has one of the world’s largest older populations and is expected to double from 2001 to 2025. By 2025, one in ten people in Bangladesh will be older adults, increasing to one in five by 2050 (6). The aging population will impact health status, the healthcare system, economic growth, labor markets, consumption, savings, investment, pension, and intergenerational transfers (6, 7). Older adults are among the poorest of the poor. In developing countries like Bangladesh, demographic and related epidemiological transitions occur in the transient socio-economic contexts (8–11). Bangladesh will need more support for the increased number of older adults (12). Increasing modernization and social change contexts confront difficulties in maintaining the honor, security, and dignity of older adults in Bangladesh (13, 14).

Most older people reside in rural areas, with widespread poor health care, economic services, and work opportunities (15, 16). Bangladesh will face a major challenge in providing adequate care and support for the increasing number of older people (15, 17). In recent times, the divorce rate in Bangladesh has been increasing dramatically (18), which will raise the number of separated and lonely aged population (16, 19). Relevant research shows that 95.0% of older people have faced health problems in Bangladesh, and most of them even face multiple health complications (16, 20, 21).

The older population is the most vulnerable group in our society due to their physical weakness, disease burden, lack of job opportunities, lack of property ownership, proper health care, etc. (21, 22). The conditions of older adult women are poorer and deplorable, especially widows and older adult women without sons are suffering more from economic and health vulnerability (16). The older population is the most vulnerable group in our society due to their physical weakness, disease burden, lack of job opportunities, lack of property ownership and proper health care, etc. (20, 22). Given the high impairment rate among older adults in Bangladesh, the link between increasing life expectancy, improved health system, and active aging situation remains inconsistent. Many of the above susceptibilities and sufferings can be reduced by improving the conditions of the determinants of active aging (6).

Enhancing active aging is a global goal aimed at improving the quality of life of older adults by addressing the issues they confront (23). World Health Organization (WHO) defined active aging as “the way of thinking and working on the process of optimizing opportunities for health, participation, and security to enhance the quality of life as people age” (24, 25). Active aging has four dimensions: health, participation, security, and employment (26, 27). Active aging includes economic and non-economic aspects of life (28). The approach has emerged in recognizing older people’s human rights and the principles of the United Nations, including participation, dignity, care, and self-fulfillment (29). Following the WHO model (29), a study used eight indicators of health dimension, three indicators of participation dimension, and seven indicators of security dimension to measure active aging in Bangladesh (30). Active Aging Index (AAI) is the average of the scores of the three dimensions, which has a minimum value of 0 and a maximum value of 1 (30).

**Figure 1:**
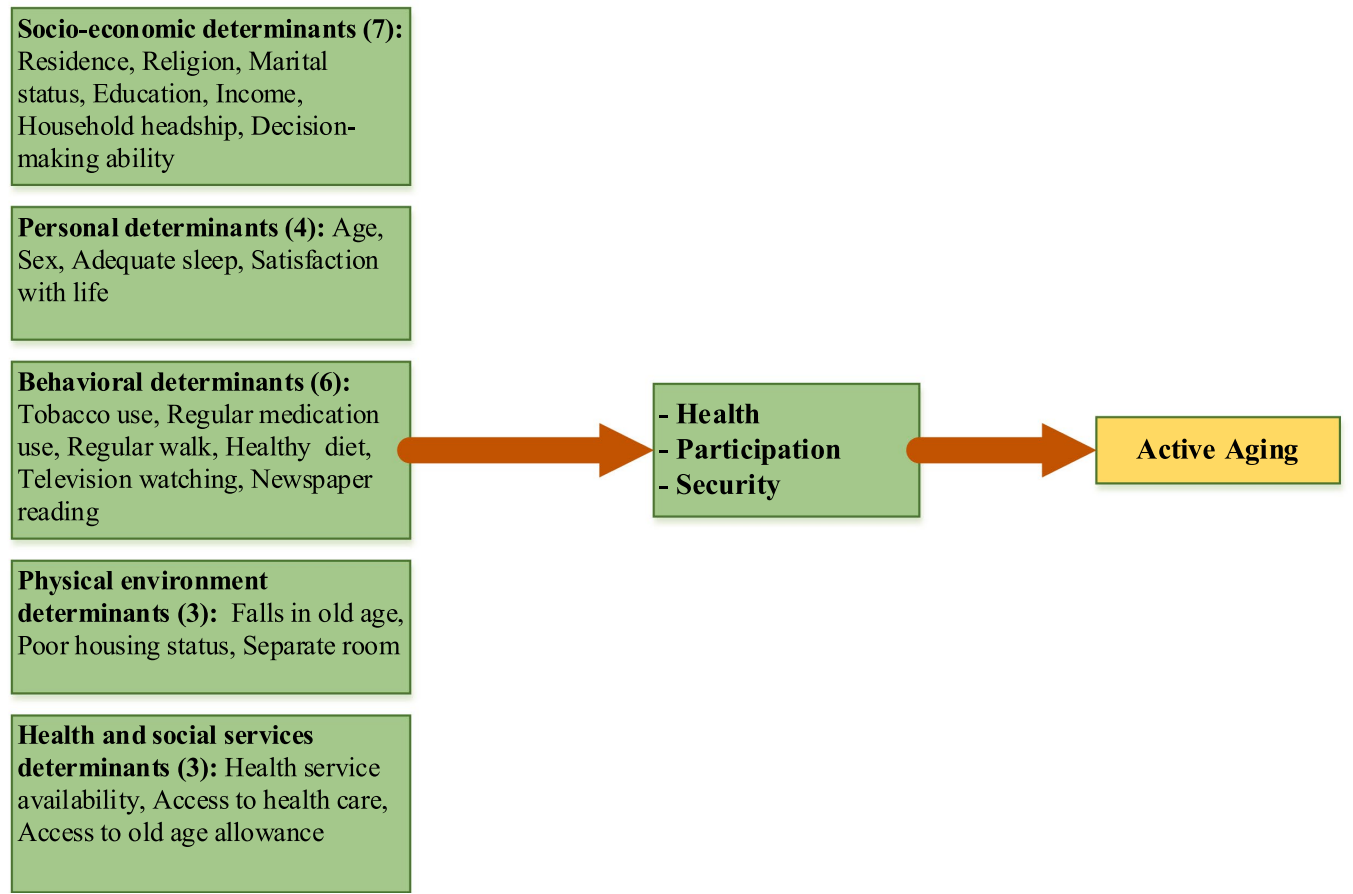
A conceptual framework, based on the WHO model (29), for active aging, including twenty-three determinants.

The higher score of the active aging index worldwide depends on a society’s socio-economic, personal, behavioral, and psychological determinants (31, 32). ‘Active aging’ results from a society’s socio-economic, cultural, and individual factors. So, based on the literature review, we have chosen the determinants of Active Aging. Considering the context and review of the above literature, a conceptual framework was developed, which included 23 determinants of active aging. The framework incorporated the demographic, socio-economic, personal, behavioral, physical environment, and health and social services contexts that determine the active aging outcome (Figure 1) (27). In these multidimensional determinants, achieving an active aging situation poses several challenges for governments, societies, and older adults regarding opportunities and contributions (33, 34).

The concepts of active aging and aging in place are leading the policies and practices of gerontology to meet the diverse needs of the aging population (35). Measuring the active aging situation has become an important research issue in Bangladesh (30), India (36), Pakistan (37), Nepal (38), and 28 European Union (EU) countries (39). This research provides insights into the potential of older adults. It can inform decision-makers to improve government policy to transform the old age group into an economically active population (40). Studies reveal that the active aging level is lower for women than men in almost all countries (41, 42). In India, inactive aging is higher than active aging (27, 36). For Bangladesh, the mean active aging score level for older men and women was 0.62 and 0.66, respectively (30). Rural and urban differentials also exist in active aging situations (43). Bangladesh’s active aging score is lower than many other EU countries (44). Considering the complex multidimensionality of the issues, little is known about the active aging situation and its determinants for older adults in Bangladesh (45, 46). The rapidly changing aging issues demand more attention from researchers and policymakers to assess the context and determinants of the active aging situation for a sustainable (1) and healthy life for the older population in Bangladesh (22, 44, 47, 48). Therefore, it is essential to conduct more research to understand the determinants affecting active aging and its dimensions in Bangladesh (10, 13).

## Methods and measures

A quantitative research approach was used in this cross-sectional study to determine the determinants of the active aging situation among people 60 years and over. Within the constraints of the research and simultaneously, to make the study more representative by capturing the more diverse scenario of the phenomenon, and with the suggestions of the sampling experts, this research selected two divisions for the study areas. Two divisions, districts and upazilas, were selected later randomly from the divisions. Cochran’s formula (49) was used to calculate the essential sample size for the required precision, confidence, and estimated population, as the total population was larger. The formula for sample size selection was:


$$n=\frac{z^{2}\times p*q}{d^{2}}\times df$$

where n = sample size; z = z value for the confidence level (e.g., 1.96 for 95% confidence level); *p* = proportion of the phenomenon under study; q = 1-*p* = 0.92; d = desired accuracy level, 0.03; df = design effect. The study adopted *p* = 0.08 as the proportion of older people in Bangladesh was 8.0% (50), and the desired level of precision in the sample was 3.0%. As multistage sampling strategies were used to select the study areas, the design effect was set at 1.65, and thus, the calculated sample size was 518. The probability proportionate sample (PPS) technique was used to distribute the number of samples between the study areas (Table 1). A list of people 60 years and over was prepared, and the sample was selected systematically after every two households.

**Table 1:** Distribution and flow chart of the randomly selected study areas and sample size.

Stage 1	Stage 2	Stage 3	Stage 4	Stage 5
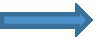	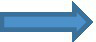	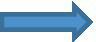	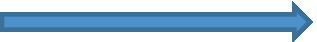	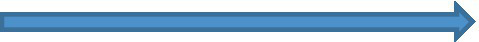
Division	District	Upazila	Union (Rural)	Ward (Urban)
Dhaka	Dhaka	Savar	Three villages of Birulia Union and 141 sample	Wards no. 6 & 7 of Birulia Paurashava and 116 sample
Rangpur	Rangpur	Pirgonj	Three villages of Raypur Union and 143 sample	Wards no 2 & 5 of Raypur Paurashava and 118 sample
			Total = 284 sample	Total = 234 sample

A semi-structured interview schedule was used for this face-to-face interview to obtain primary data on the socio-economic, personal, behavioral, lifestyle, and health-related twenty-three determinants of active aging. Ethical clearance was obtained from the concerned body, and informed consent was obtained from the respondents. With the help of three trained research assistants (RAs), data were collected from the respondents aged 60 years and over from October to December 2019. The researcher, in person, trained the research assistants (RA) about the research objectives, questions, concepts, wording of the questions, and study settings in detail. RAs participated in pretesting the questionnaire. The RAs stayed in the field with the researcher and collected data through face-to-face interviews. Data were analyzed using univariate and multivariate statistics. The included independent variables for the study (Figure 1) were quantitative, and responses were either dichotomous or continuous, and the dependent variable ‘active aging’ was continuous. The respondents’ responses for each indicator in each dimension of active aging were added to create the composite index for each active aging dimension (30). A multiple linear regression technique was used to assess the effect of the determinants on active aging. SPSS version 18.0 was used to analyze the data. More details of the construction of the AAI can be found in the authors’ earlier paper on Active Aging (30).

### Results of the study

Table 2 shows the socio-demographic characteristics of the respondents. Five hundred eighteen respondents were interviewed; 265 were men, and 253 were women. The majority (64.5%) of the older adults were young-old (60–69 years), and 23.5% were middle-old (70–79 years). About 91.0% of the respondents were Muslim, 7.9% were Hindu, and 1.2% were Christian. About 55.0% of the respondents were from rural areas, 45.2% were from urban areas, and 71.2% were married. About 59.0% of the respondents had no formal education, 61.6% had limited income, 67.0% were household heads, and 73.6% were involved in household decision-making.

**Table 2:** Socio-demographic characteristics of the respondents.

Characteristics	Frequency	Percentage
Age		
Young-old (60–69)	334	64.5
Middle-old (70–79)	122	23.5
Old-old (80^+^)	62	12.0
Sex		
Male	265	51.2
Female	253	48.8
Religion		
Muslim	471	90.9
Hindu	41	7.9
Christian	6	1.2
Place of residence		
Rural	284	54.8
Urban	234	45.2
Current marital status		
Currently married	370	71.4
Unmarried	4	0.8
Widowed	142	27.4
Divorced	2	0.4
Educational status		
No education	306	59.1
Primary education	119	23.0
Secondary education	67	12.9
Higher secondary or above	26	5.0
Income		
Have income	319	61.6
No income	199	38.4
Household headship		
Yes (still, he/she is the household head)	347	67.0
No	171	33.0
Decision-making ability		
Yes	381	73.6
No	137	26.4

Source: Primary data analysis.

Following the physical environment, 68.5% of respondents opined that they had better housing status, 86.1% had a separate room, and 27.4% perceived that they had fallen in old age. Regarding health and social services, 60.0% said that health services were available but not accessible (52.5%). Only 15.0% of the respondents reported getting an old age allowance (Table 3).

**Table 3:** Physical environment, health, and social services characteristics of the respondents.

Characteristics	Yes		No	
	Frequency	Percentage	Frequency	Percentage
Better housing status	355	68.5	163	31.5
Separate room	446	86.1	72	13.9
Falls in old age	142	27.4	376	72.6
Health service availability	311	60.0	207	40.0
Health service accessibility	246	47.5	272	52.5
Get an old age allowance	78	15.1	440	84.9

Source: Primary data analysis.

### Determinants of active aging situation

Table 4 shows that males (0.64), young-old (60–69) (0.61), those who are currently married (0.61), residing in urban (0.63) areas, have higher education (0.63), access to income (0.63), still household heads (0.64), and decision-making capacity (0.64) had a higher mean score of AAI than other groups.

**Table 4:** Active Aging Index (AAI) by socio-demographic characteristics of the respondents.

Characteristics	Frequency (%)	Active Ageing Index (AAI) (Mean Value)*	*P* value
Sex			
Male	265 (51.2)	0.64	0.000
Female	253 (48.2)	0.53	
Age			
Young-old (60–69)	334 (64.5)	0.61	0.000
Middle-old (70–79)	122 (23.5)	0.57	
Old-old (80+)	62 (12.0)	0.51	
Marital status			
Married	370 (71.4)	0.61	0.000
Others	148 (28.6)	0.54	
Residence			
Urban	234 (45.2)	0.63	0.000
Rural	284 (54.8)	0.55	
Religion			
Muslim	471 (90.9)	0.57	0.254
Others	47 (9.1)	0.62	
Education			
Yes	212 (40.9)	0.63	0.000
No	306 (59.1)	0.56	
Income			
Have income	319 (61.6)	0.63	0.000
No income	199 (38.4)	0.51	
Household headship			
Yes	347 (67.0)	0.64	0.000
No	171 (33.0)	0.56	
Decision-making ability			
Yes	381 (73.6)	0.64	0.000
No	137 (26.4)	0.55	

Source: Primary data analysis; * (authors’ earlier paper on Active Aging) (30).

Around 60.0% of the respondents were satisfied with their lives, 37.5% had sufficient sleep (eight hours or more), 45.6% could not consume a balanced diet, and only 24.3% had regular walking as exercise. Most of the older adults (65.0%) were used to smokeless tobacco consumption. Following leisure activities, 45.4% used to watch television, and 8.3% had the habit of newspaper reading. Only 41.1% conform to regular medicine use. The mean scores of active aging were found to be higher among the respondents who had higher personal factors such as perceived life satisfaction (0.64) and sleeping hours more than 8 h (0.65) compared to those who were not satisfied (0.59) and slept less than 8 h (0.59). Similar to personal characteristics, mean scores of active aging were found to be higher for behavioral traits such as older adults having a balanced diet (0.64), practicing regular morning walking (0.65), avoiding tobacco consumption (0.64), watching television (0.65) and reading a newspaper as leisure activities (0.73), regular medicine use (0.59) compared to who did not (Table 5).

**Table 5:** Respondents by the determinants (personal and behavioral) of the Active Aging Index (AAI).

Determinants of AAI	Frequency (%)	Active Aging Index (AAI) (Mean value)*	*p* value
Perceived life satisfaction			
Dissatisfied	115 (22.2)	0.59	0.000
I am neither satisfied nor dissatisfied	90 (17.4)	0.57	
Satisfied	313 (60.4)	0.64	
Duration of sleep			
<8 h	322 (62.4)	0.59	0.000
>8 h	194 (37.6)	0.65	
Balanced diet consumption			
Yes	282 (54.4)	0.64	0.000
No	236 (45.6)	0.59	
Regular walking as exercise			
Yes	126 (24.3)	0.65	0.001
No	392 (75.7)	0.60	
Smokeless tobacco consumption			
Yes	336 (64.9)	0.60	0.014
No	182 (35.1)	0.64	
TV watching as a leisure status			
Yes	235 (45.4)	0.65	0.000
No	283 (54.6)	0.58	
Newspaper reading			
Yes	43 (8.3)	0.73	0.000
No	474 (91.7)	0.60	
Regular medication use			
Yes	213 (41.1)	0.59	0.004
No	305 (58.9)	0.63	

Source: Primary data analysis; * (authors’ earlier paper on Active Aging) (30).

Respondents’ active aging condition varied by physical environmental characteristics, including having better, adequate housing status and separate rooms, not being perceived as falling into older age, having availability and accessibility to health services, and getting an old age allowance (Table 6).

**Table 6:** Physical environment, health, and social services characteristics of the respondents by AAI.

Determinants	Frequency (%)	Active Aging Index (Mean Value)*	*p* value
Better housing status			
Yes	355 (68.5)	0.63	0.014
No	163 (31.5)	0.59	
Separate room			
Yes	446 (86.1)	0.63	0.000
No	72 (13.9)	0.55	
Falls in old age			
Yes	142 (27.4)	0.58	0.000
No	376 (72.6)	0.63	
Health service availability			
Yes	311 (60.0)	0.63	0.002
No	207 (40.0)	0.59	
Health service accessibility			
Yes	246 (47.5)	0.64	0.000
No	272 (52.5)	0.59	
Get an old age allowance			
Yes	78 (15.0)	0.58	0.035
No	440 (85.0)	0.62	

Source: Primary data analysis; * (authors’ earlier paper on Active Aging) (30).

**Figure 2:**
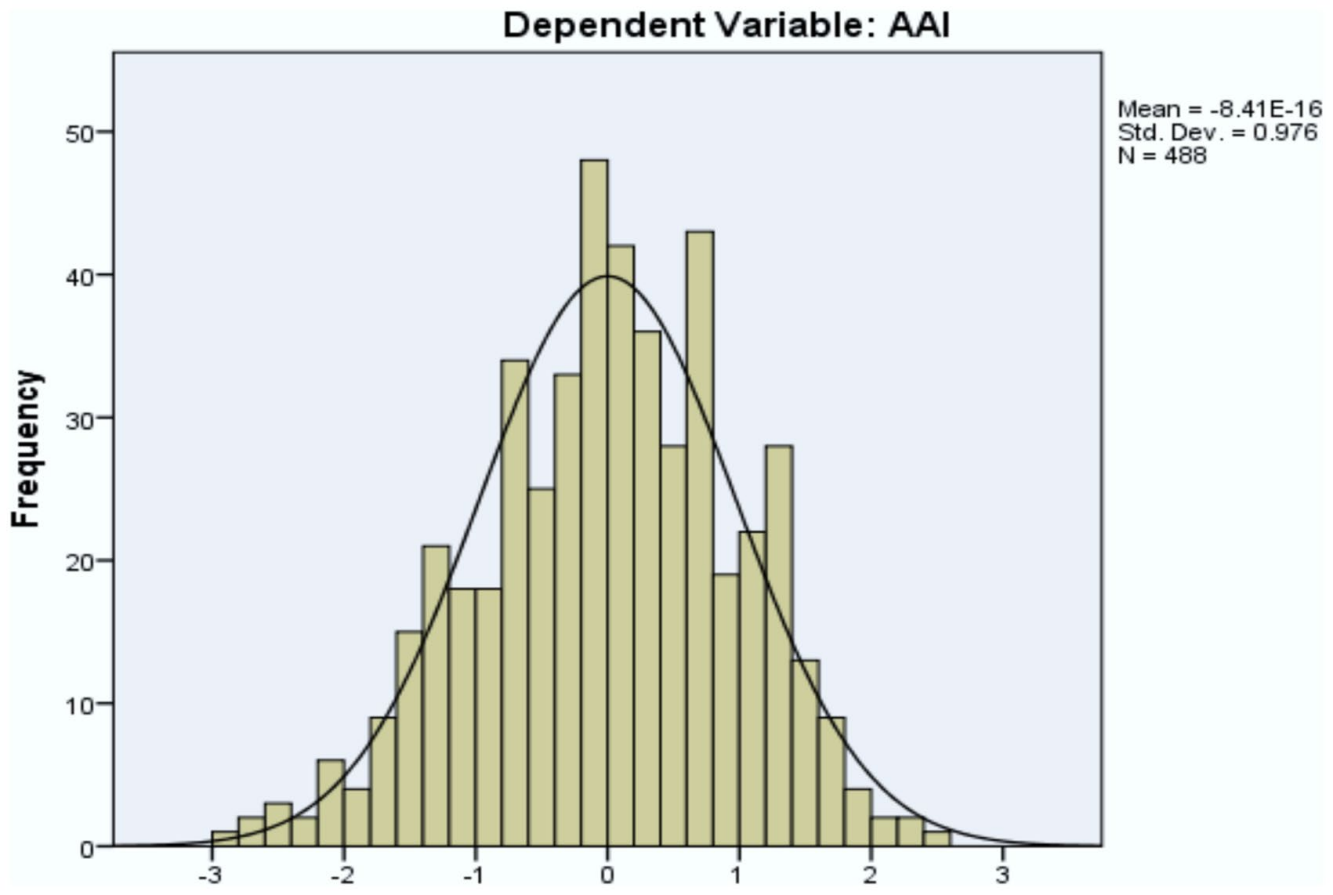
Regression standardized residual. Source: Primary data analysis.

**Figure 3:**
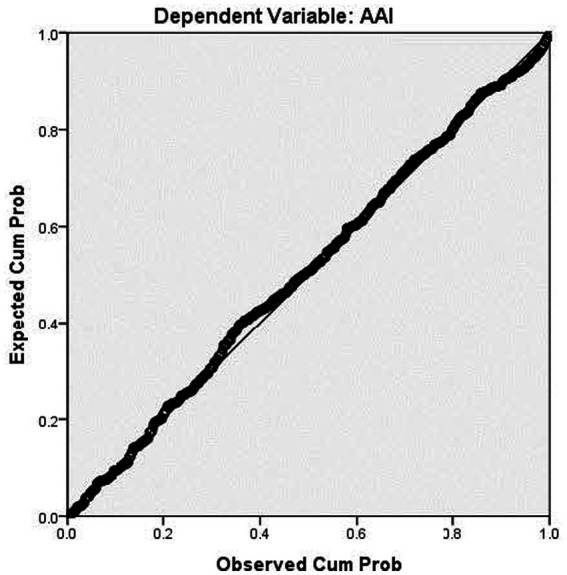
Normal P–P plot regression standardized residual. Source: Primary data analysis.

Figure 2 presented the regression standardized residual, and Figure 3 presented the normal P-P Plot regression standardized residual.

The result of the multiple linear regression model shows that even after controlling all the variables, marital status (AOR = 0.197, 95% CI: 0.018–0.105), income (AOR = 0.121, 95% CI: 0.002—0.042), decision-making ability (AOR = 0.110, 95% CI: 0.001—0.074), regular walking/physical exercise (AOR = 0.120, 95% CI: 0.005—0.067), smokeless tobacco consumption (AOR = −0.110, 95% CI: −0.061— −0.005), newspaper reading (AOR = 0.113, 95% CI: 0.004—0.094) as leisure status, use of medication (AOR = −0.172, 95% CI: −0.076—−0.023), and health service accessibility (AOR = 0.161, 95% CI: 0.015—0.076) had significant effect on the mean score of active aging (Table 7).

**Table 7:** Determinants associated with active aging.

Characteristics	Adjusted odds	95% CI	
		Lower bound	Upper bound
Socioeco-demographic determinants			
Sex: Male, *[Ref: Female]*	0.010	−0.042	0.048
Age: 1 year as a unit	−0.057	−0.003	0.001
Marital status: Married *[Ref: Others]*	0.197^**^	0.018	0.105
Residence: Urban, *[Ref: Rural]*	−0.044	−0.043	0.018
Education: Years of study	0.014	−0.003	0.004
Religion: Muslim, *[Ref: others]*	−0.158^**^	−0.125	−0.029
Income: Yes, *[Ref: No]*	0.121^*^	0.002	0.042
Household headship: Yes, *[Ref: No]*	−0.070	−0.067	0.017
Decision-making ability: Yes, *[Ref: No]*	0.110^**^	0.001	0.074
Personal and behavioral determinants			
Satisfaction in life: Dissatisfied in life, *[Ref: Satisfied]*	−0.075	−0.058	0.006
Adequate Sleep: Sleeping 8 h+: *[Ref: <8 h]*	0.076	−0.004	0.048
Balanced diet consumption: Yes, *[Ref: No]*	0.092	−0.005	0.057
Regular walking/physical exercise: Yes, *[Ref: No]*	0.120^**^	0.005	0.067
Smokeless tobacco consumption: Yes, *[Ref: No]*	−0.110^**^	−0.061	−0.005
TV watching: Yes, *[Ref: No]*	−0.023	−0.035	0.022
Newspaper reading: Yes, *[Ref: No]*	0.113^**^	0.004	0.094
Physical Environment, health, and social services determinants			
Regular medication use: Yes, *[Ref: No]*	−0.172^**^	−0.076	−0.023
Better housing status: Yes, *[Ref: No]*	0.089	−0.005	0.059
Separate room: Yes, *[Ref: No]*	0.034	−0.028	0.056
Falls in old age: Yes, *[Ref: No]*	−0.068	−0.054	0.009
Reported health service availability: Yes, *[Ref: No]*	0.015	−0.024	0.033
Reported health service accessibility: Yes, *[Ref: No]*	0.161^**^	0.015	0.076
Get an old age allowance: Yes, *[Ref: No]*	−0.075	−0.062	0.012
Constant (unadjusted coefficient) = 0.678	0.678	0.508	0.884
Goodness of fit R^2^ = 0.450; adjusted R^2^ = 0.406			

Source: Primary data analysis; (CI: Confidence Interval. *p value < 0.10. ** *p*-value < 0.05).

## Discussion

This research is one of the very first of its kind in Bangladesh, where behavioral, health, and physical determinants were assessed, and the effects of different socio-demographic, economic, health, personal, behavioral, and physical determinants were evaluated to know the active aging situation of the country. Developed and developing countries have insisted on studying active and healthy aging situations. However, there is a scarcity of research on active aging in Bangladesh (46). The calculated active aging score for rural males and females was 0.65 and 0.58, respectively. For urban males and females, it was 0.65 and 0.57, respectively (46), which is very low compared to many European and East Asian countries (44). The construction of active aging using WHO models and identifying the effects of the determinants on the active aging situation was a unique initiative in Bangladesh, which was earlier applied to other countries’ situations (23, 42). The constructed determinants framework for assessing the active aging situation was new for the country.

The active aging score is influenced by various individual, familial, societal, and national levels determinants (29). In Bangladesh, demographic and socio-economic factors also differentiate active aging, and the findings showed that active aging was higher among young-old people, corroborated by another study (51). Following the findings, it was found that the active aging score was higher among males than females (52), older adults living in urban areas (23), and those who had income and family decision-making abilities (53). Similarly, personal and behavioral characteristics were linked to active aging situations, and the findings were also consistent with those of other studies (54–57). The score for active aging also differed regarding the respondents’ physical environment, health, and social service characteristics, corroborating another study (57).

Considering the global impact of population aging, the United Nations General Assembly declared 2021–2030 as the ‘Decade of Healthy Ageing’ (58, 59). The focus on active aging was growing and was included in the healthy aging umbrella with other important issues of this rights-based approach (60), like no one left behind (61) and social safety net programs for all (62). Active aging issues are broadly circulated in academic, social, political, and media (62). As part of the concern, the findings show insufficient research and contextual evidence on the reality of active aging in Bangladesh. This primary research and its findings reveal the first evidence of systematically identifying the reality of the determinants of the active aging situation in Bangladesh. The study identified, field-tested, and found the significant effects of nine determinants on the active aging index. These determinants are marital status, income, decision-making ability, regular walking/physical exercise, smokeless tobacco consumption, newspaper reading as leisure status, use of medication, and health service accessibility, and they have similarities with the aging situation of other countries (63). Recognition of all the factors may create opportunities for researchers and policy makers to improve active aging situations across different communities and nations.

Findings showed that many socio-demographic, economic, personal, and behavioral determinants are interrelated (31). For example, higher income and education may affect diet, physical exercise, and safe living (45). This inter-relationship between the determinants needs to be accounted for when measuring active aging situations (45). People may value a particular factor more in a given context and culture. It implied that the active aging situation is not isolated from context or static (31). Therefore, a successful measure of active aging situations needs to assess all the known determinants and comprehend the value and importance the individual attaches to each determinant at the individual and community levels (64, 65). The study gained its strengths by introducing an initiative to identify the effect of the determinants on the active aging domains and developing tools for field data collection, which will significantly help future researchers (32).

The study also framed future research directions for assessing active aging situations. Although this study’s cross-sectional methodology allowed for describing relationships between numerous indicators and active aging scores, causal implications cannot be reached. A longitudinal study would be more instrumental in assessing the factors influencing the active aging situation. More qualitative research helps better understand the aspects of active aging. This study did not include older people with psychiatric problems, but they should be included in future studies. Considering the context and reality of a society, different instruments may be used to measure the active aging situation (57).

This study included respondents from only selected rural and urban areas. Thus, further research may be required to generalize the other parts. Furthermore, applying and measuring the WHO policy framework of active aging in Bangladesh to analyze the determinants is quite complex and challenging to quantify in a direct measure. Consequently, some proxy indicators were taken into account.

## Conclusion

From the perspective of the continuous growth of older people, ensuring active aging is critical. Determinants of active aging should be considered to enhance the overall wellbeing of older people. Identified determinants can guide policymakers on where to focus and how strategies and action plans must be revisited. Immediate attention is required to improve every aspect of active aging. Based on the identified determining factors of active aging, appropriate programs should be formulated and implemented to increase future active aging levels of the country and countries with similar socio-economic settings. Countries should regularly assess the situation of the active aging determinants to ensure the overall level of active aging and help achieve transformative, inclusive, and sustainable development outcomes. Future studies should include a broader geographic range and a larger, more diverse sample for greater generalizability. Moreover, a follow-up longitudinal study could explore causal relationships and the evolution of active aging determinants over time. Future research may also incorporate older adults with psychiatric and cognitive impairments to provide a more holistic perspective. Actionable strategies such as income-generation programs or community-based health interventions should be taken for the improvement of AAI. The study findings may serve as a benchmark for future research on active aging in similar socio-economic settings.

## Data Availability

The raw data supporting the conclusions of this article will be made available by the authors, without undue reservation.
